# Detection of Replication Competent Lentivirus Using a qPCR Assay for VSV-G

**DOI:** 10.1016/j.omtm.2017.09.001

**Published:** 2017-09-21

**Authors:** Lindsey M. Skrdlant, Randall J. Armstrong, Brett M. Keidaisch, Mario F. Lorente, David L. DiGiusto

**Affiliations:** 1 Laboratory for Cell and Gene Medicine, Department of Pediatrics, Stanford University School of Medicine, Palo Alto, CA 94304, USA

**Keywords:** PCR, lentivirus, WPRE, CAR T

## Abstract

Lentiviral vectors are a common tool used to introduce new and corrected genes into cell therapy products for treatment of human diseases. Although lentiviral vectors are ideal for delivery and stable integration of genes of interest into the host cell genome, they potentially pose risks to human health, such as integration-mediated transformation and generation of a replication competent lentivirus (RCL) capable of infecting non-target cells. In consideration of the latter risk, all cell-based products modified by lentiviral vectors and intended for patient use must be tested for RCL prior to treatment of the patient. Current Food and Drug Administration (FDA) guidelines recommend use of cell-based assays to this end, which can take up to 6 weeks for results. However, qPCR-based assays are a quick alternative for rapid assessment of RCL in products intended for fresh infusion. We describe here the development and qualification of a qPCR assay based on detection of envelope gene sequences (vesicular stomatitis virus G glycoprotein [*VSV-G*]) for RCL in accordance with Minimum Information for Publication of Quantitative Real-Time PCR Experiments (MIQE) guidelines. Our results demonstrate the sensitivity, linearity, specificity, and reproducibility of detection of *VSV-G* sequences, with a low false-positive rate. These procedures are currently being used in our phase 1 clinical investigations.

## Introduction

Viral vectors are a common tool for introducing new or corrected genes into human cells for production of cellular therapy products. Chimeric antigen receptor T (CAR T) cells require retrovirus or lentivirus to introduce the CAR gene into activated T cells.^1^, ^2^, ^3^ Lentiviral vectors have also been used for the treatment of hereditary disorders, such as Wiskott-Aldrich syndrome and severe combined immunodeficiency disorder (SCID).^4^, ^5^ Viral vectors are preferred for these therapies due to their ability to directly insert genes of interest into the host genome, allowing for stable long-term production of the gene of interest in both the originally transduced cells and their progeny.^6^, ^7^, ^8^, ^9^, ^10^ However, the use of viral vectors to introduce genes into cells is not without certain safety concerns, among which is the development of replication competent virus during the production of the viral vectors.^7^, ^11^, ^12^, ^13^

First- and second-generation retroviral vectors have previously had issues with recombination, leading to replication competent virus causing lymphomas in primate models^6^, ^7^, ^11^, ^12^, ^13^ and insertional mutagenesis causing lymphomas in human patients.^14^, ^15^, ^16^ As a result, the Food and Drug Administration (FDA) requires all cell products transduced with retroviral vectors to be tested for replication competent virus prior to treatment of the patient with the product.^11^, ^17^ Although no specific guidance for lentiviral vectors has been provided, cell products transduced with lentivirus fall under the same recommendations as retrovirus.^11^, ^17^, ^18^ The FDA currently recommends the use of a cell-based assay;^11^, ^17^, ^18^ however, many phase I/phase II clinical trials of novel cellular therapies require the infusion of fresh cells, which does not allow completion of a full cell-based replication competent lentivirus (RCL) assay (up to 6 weeks) prior to infusion. For this reason, a quantitative PCR-based assay would be an ideal acceptable method for a rapid assessment of RCL prior to a fresh product release.^11^, ^17^, ^18^

Previous qPCR-based tests for RCL in second-generation lentiviral vectors have targeted detection of backbone elements, such as *tat*, *gag*, and *pol*, and pseudotyping envelope proteins, such as vesicular stomatitis virus G glycoprotein (VSV-G).^19^, ^20^ RCL of third-generation lentiviral vectors has never been observed,^18^, ^20^, ^21^ but the most likely scenario for formation of RCL would be through recombination of the transfer plasmid with the supporting packaging plasmids.^22^ *Tat* gene is no longer present in third-generation lentiviral vectors.^8^, ^10^, ^13^, ^23^ Gag-pol sequences are more variable than envelope sequences,^24^, ^25^, ^26^, ^27^ and a previous study has shown that although high levels of psi-gag recombinants are often observed in packaging cells, they did not correlate with RCL occurrence based on a cell-based RCL assay.^20^ Therefore, the envelope gene (*VSV-G* in our case) is the most suitable target for detecting RCL because incorporation of an envelope gene sequence would be required to generate a replication competent virus and envelope genes are the best suited for reliable detection.

This report outlines the development of a qPCR assay to detect *VSV-G* DNA in the presence of human genomic DNA. This assay is consistent with the detection of *VSV-G* in the same background as would be present in cell therapy product samples and the Minimum Information for Publication of Quantitative Real-Time PCR Experiments (MIQE) guidelines.^28^ Finally, we show that the method of sample preparation chosen is sufficient to remove background *VSV-G* DNA from transduced cell therapy products and reduce the detection of false positive replicates.

## Results

**Figure 1:**
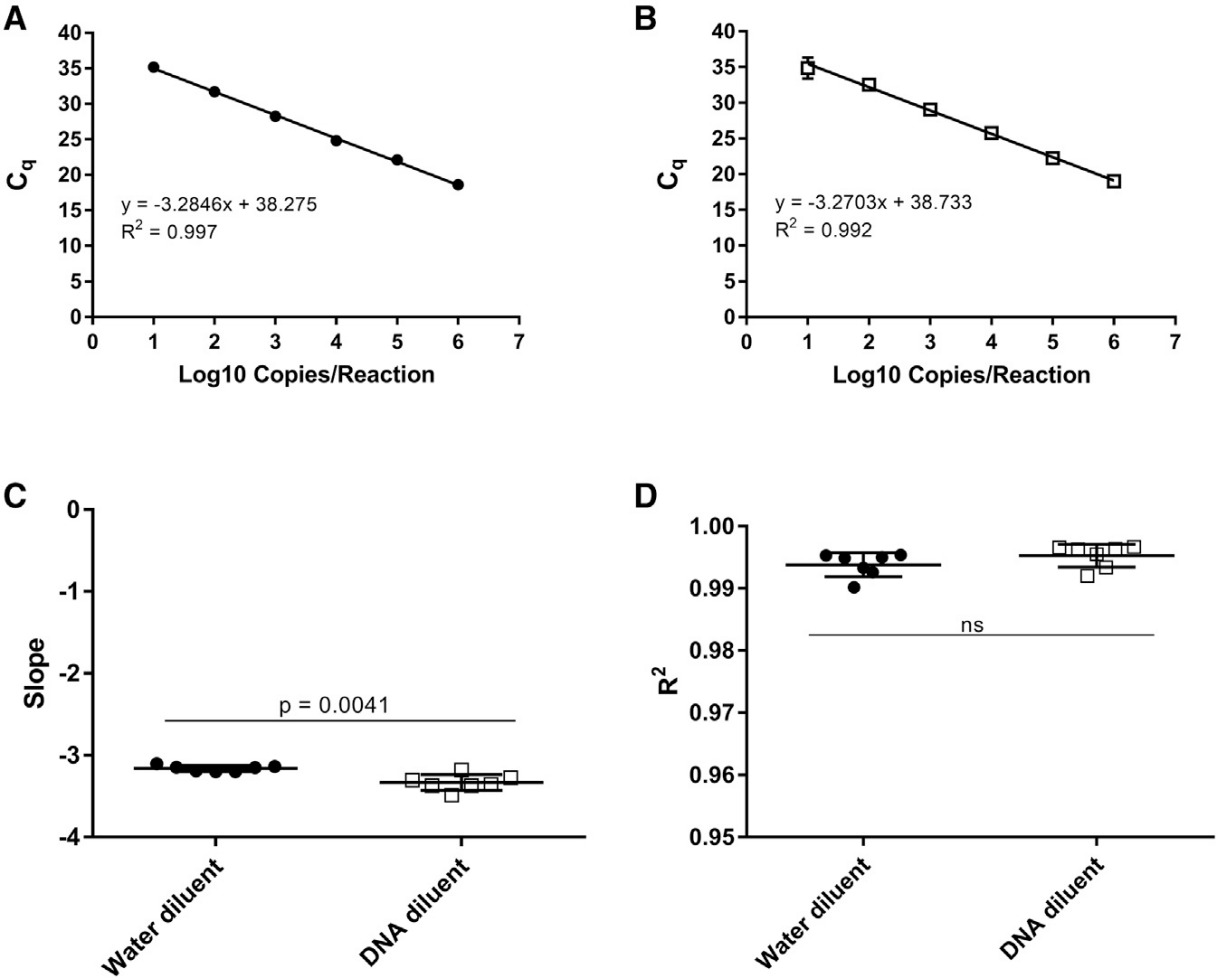
Standard Curve Comparison (A and B) Representative standard curves for *VSV-G* using nuclease-free water as the diluent (A) or control human DNA as the diluent (B). (C) Average slopes for *VSV-G* standard curves using nuclease-free water or control human DNA as the diluent (n = 7). (D) Average R^2^ values for *VSV-G* standard curves using nuclease-free water or control human DNA as the diluent (n = 7). Error bars represent SD.

For our initial assessment of a qPCR assay for RCL detection, we created a 10-fold dilution series (10^1^ to 10^6^ copies) of *VSV-G* plasmid in nuclease-free water as a standard curve. To assess the effect of genomic DNA on PCR detection, we created a second set of standards that included human genomic DNA at a concentration of 8 ng/μL (100 ng/reaction) at all *VSV-G* concentrations tested. We evaluated PCR amplification of *VSV-G* sequences in both standard curve formulations under identical reaction conditions. Representative results from PCR amplification using each of these two standard curve types are shown in Figures 1A and 1B. Standard curves with nuclease-free water as the diluent produced a mean slope of −3.1599 ± 0.0684, whereas standard curves with control human DNA as the diluent produced a mean slope of −3.3322 ± 0.1771 (Figure 1C). Although the slopes for these groups were statistically significantly different (p = 0.0041), they are within 10% of the ideal slope of −3.32 that is expected in a theoretical qPCR standard curve assay with 100% amplification efficiency and thus acceptable according to MIQE guidelines. Mean R^2^ values for each were 0.9938 ± 0.0036, with the lowest value of 0.9902 for standard curves with the nuclease-free water diluent, and 0.9953 ± 0.0034, with the lowest value of 0.9920 for standard curves with control human DNA as the diluent (Figure 1D). These data show that for a 10-fold standard curve, there is no significant difference in assay linearity between using nuclease-free water or control human DNA as the diluent.

**Figure 2:**
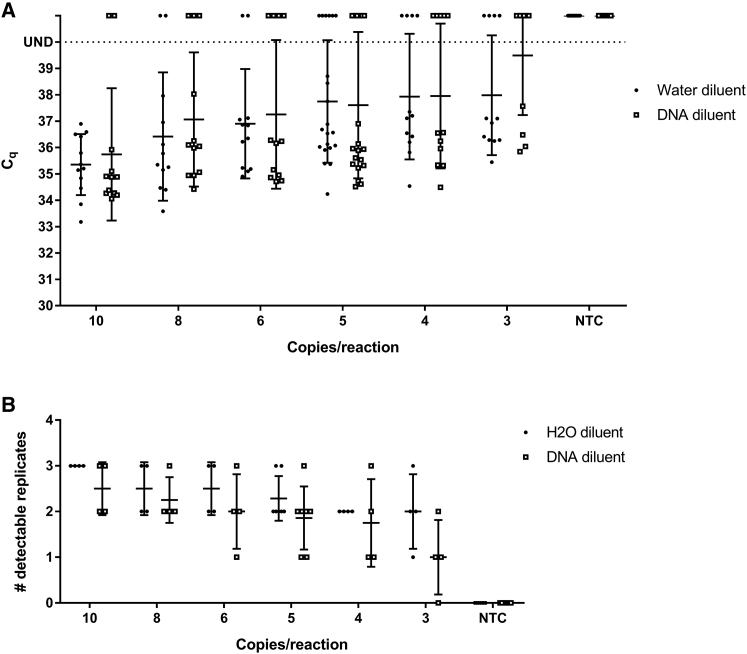
Limit of Detection for *VSV-G* in Nuclease-free Water and Control Human DNA (A) Quantification cycle (C_q_) values for 10, 8, 6, 5, 4, and 3 copies/reaction and NTC. UND, undetectable; sample detection failed to reach the threshold of detection in 40 cycles (n = 4; 3 replicates per assay). (B) Number out of 3 replicates detectable within 40 reaction cycles for 10, 8, 6, 5, 4, and 3 copies/reaction and NTCs (n = 4). Error bars represent SD.

*VSV-G* detection should be sensitive enough to reproducibly identify samples with low levels of RCL without misidentifying samples with PCR signals that arise spuriously. Therefore, the limit of detection (LOD) and false positive rate was established for each standard curve type to define the lowest number of *VSV-G* DNA copies that can be accurately and reproducibly measured with this technique. Test articles were created containing 10, 8, 6, 5, 4, or 3 copies of *VSV-G* DNA sequence per reaction to represent the expected and practical limits of sensitivity for these reactions. The frequency of detection of *VSV-G* sequences spiked into nuclease-free water is 100% for 10 copies, 83.33% for 8 copies and 6 copies, 76.19% for 5 copies, and 66.67% for both 4 copies and 3 copies (Figure 2; Table S1). However, the frequency of detection of *VSV-G* DNA spiked into control human DNA was 83.33% for 10 copies, 75% for 8 copies, 66.67% for 6 copies, 61.90% for 5 copies, 58.33% for 4 copies, and 33.33% for 3 copies (Figure 2; Table S1). Although the C_q_ values for *VSV-G* in a genomic DNA background were not statistically different from those with *VSV-G* DNA in a background of nuclease-free water, only the detection of 10 copies of *VSV-G* in nuclease-free water falls within the 95% positive detection range required by the MIQE guidelines to be defined as the LOD. This shows that detection of *VSV-G* DNA in a nuclease-free water background is more reproducible at low copy numbers than detection in a sample containing human genomic DNA.

**Figure 3:**
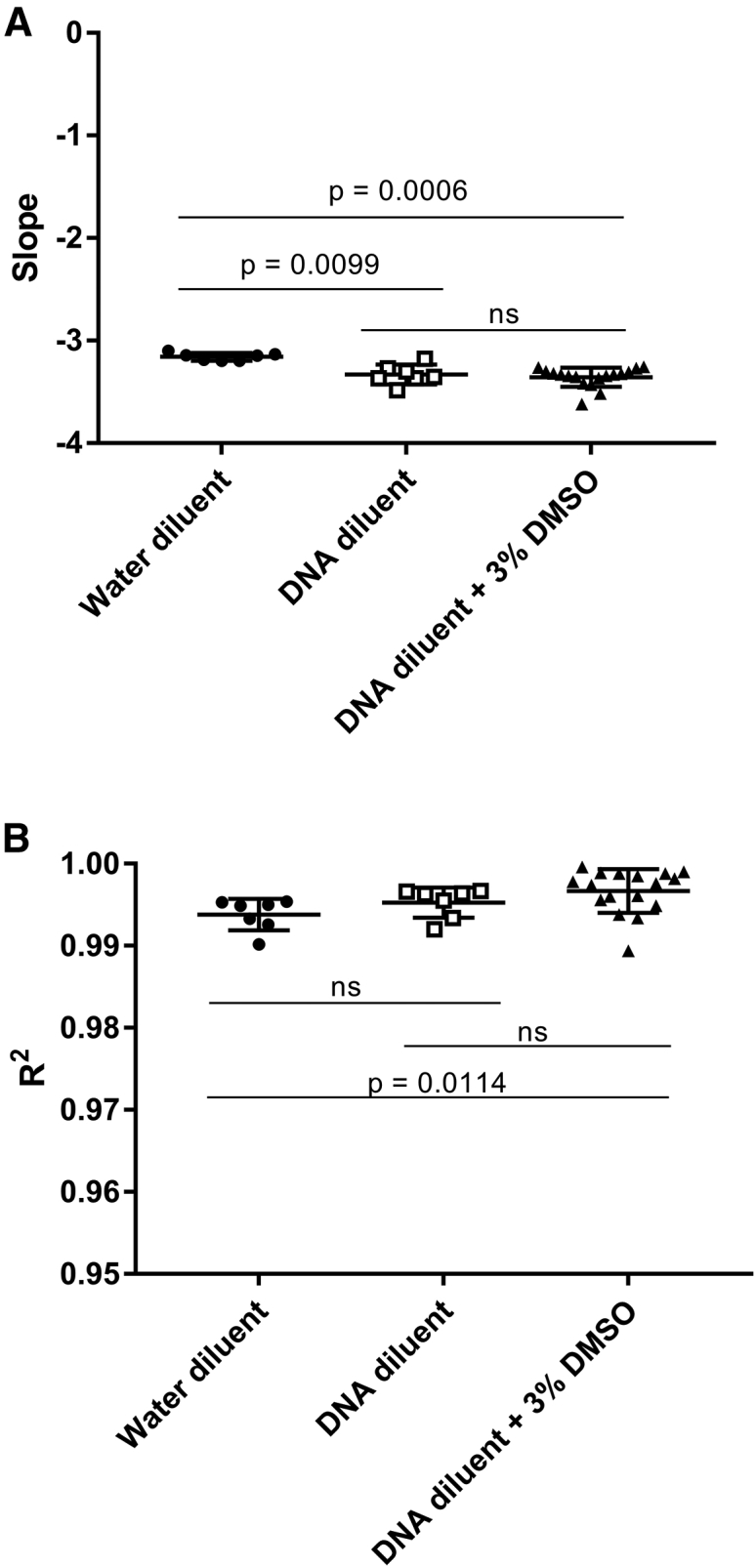
Standard Curve Comparison with DMSO Standard Curves (A) Average slopes for *VSV-G* standard curves using nuclease-free water (n = 7; repeated from Figure 1 for comparison), control human DNA (n = 7; repeated from Figure 1 for comparison), or control human DNA with a final 3% (v/v) DMSO reaction concentration (n = 16) as the diluent. (B) Average R^2^ values for *VSV-G* standard curves using nuclease-free water (n = 7; repeated from Figure 1 for comparison), control human DNA (n = 7; repeated from Figure 1 for comparison), or control human DNA with a final 3% (v/v) DMSO reaction concentration (n = 16) as the diluent. Error bars represent SD.

**Figure 4:**
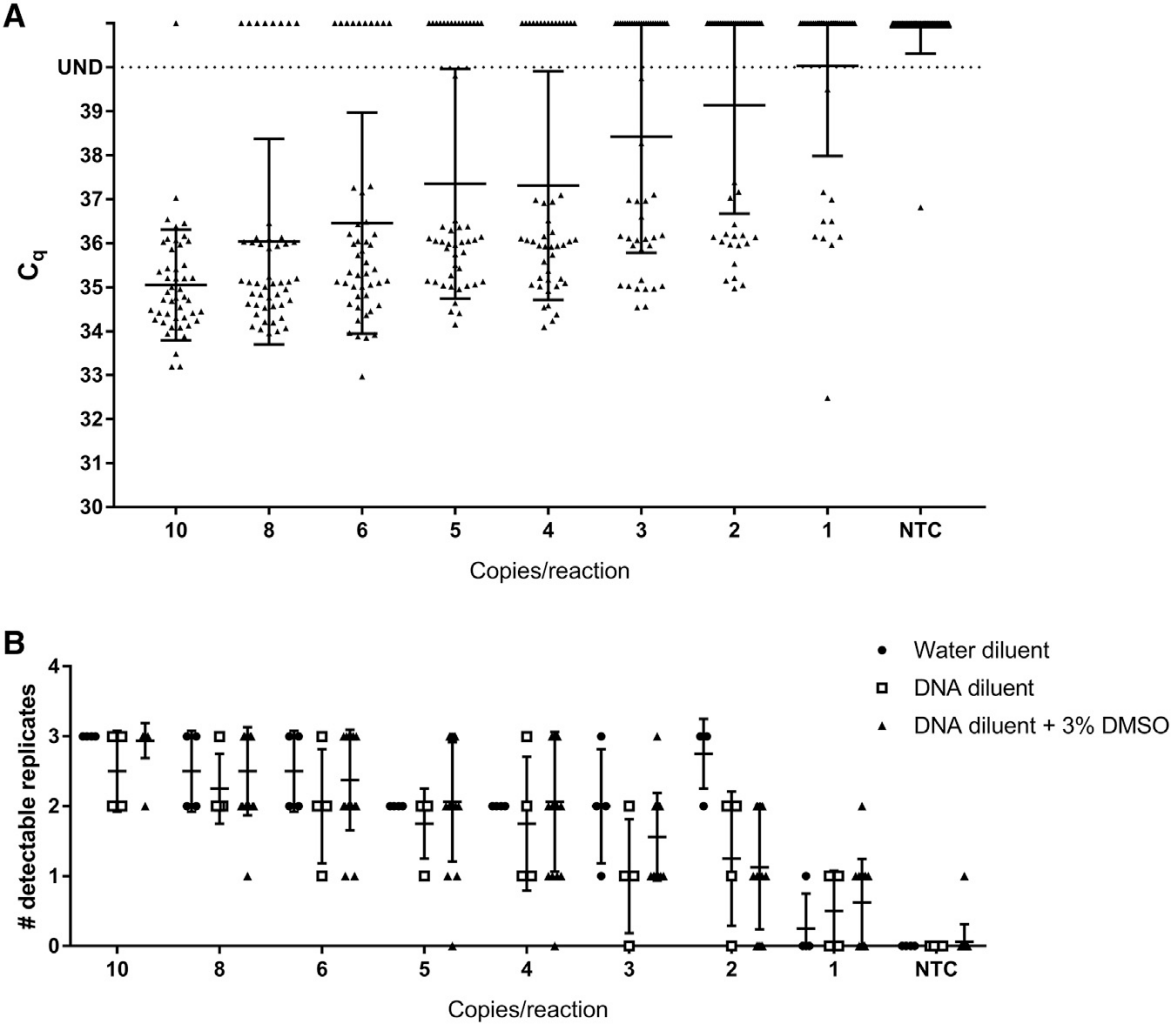
Limit of Detection for *VSV-G* in Control Human DNA with a 3% v/v Final Concentration of DMSO in the Reaction (A) C_q_ values for 10, 8, 6, 5, 4, and 3 copies/reaction and NTC. UND, undetectable; sample detection failed to reach the threshold of detection in 40 cycles (n = 16; 3 replicates per assay). (B) Number of 3 replicates detectable within 40 reaction cycles for 10, 8, 6, 5, 4, and 3 copies/reaction and NTCs (n = 16). Data from Figure 2B are included for comparison. Error bars represent SD.

DMSO has previously been shown to improve both the specificity and sensitivity of qPCR.^29^ It was tested in the context of *VSV-G* detection by qPCR, as described above in samples containing human genomic DNA. DMSO at a final concentration of 3% in the reaction volume showed improvement in the detection of a couple of samples of *VSV-G* spiked into human genomic DNA (data not shown), so we tested 3% DMSO in a full standard curve assay, with low-copy spiked in *VSV-G* controls for limit of detection determination. The use of 3% DMSO in the PCR reaction was then tested in a full *VSV-G* qPCR assay for a 10-fold dilution series standard curve and quality control (QC)-positive control samples. This assay was repeated for a total of 16 biological replicates and compared to the previous data in Figure 2 for nuclease-free water and control human DNA alone as the diluents for the standard curve. Standard curves with control human DNA as the diluent and a 3% (v/v) final concentration of DMSO produced a mean slope of −3.3422 ± 0.2354 (Figure 3A), which is near the ideal of a slope of −3.32 for a 100% efficient PCR reaction and not statistically different than the slopes observed for standard curves in a DNA background alone. Mean R^2^ values were 0.9957 ± 0.0059, with the lowest value of 0.9894 for standard curves with control human DNA in the diluent and a 3% (v/v) final concentration of DMSO (Figure 3B). Although not statistically different than the R^2^ for DNA alone, this range was statistically closer to true linearity (R^2^ = 1) than standard curves with nuclease-free water. The limit of detection was also assayed in the new reaction conditions. The frequency of detection in control human DNA with 3% (v/v) DMSO is 97.92% for 10 copies, 83.33% for 8 copies, 79.17% for 6 copies, 68.75% for 5 copies and 4 copies, and 52.08% for 3 copies (Figure 4; Table S2). These data support the use of DMSO to improve the reproducibility of detection of low levels of *VSV-G* DNA in the presence of genomic DNA and approximates that seen when assaying *VSV-G* DNA in nuclease-free water. The standard curve assay was also performed independently by three operators to assess inter-operator variability. These assays all fell within the acceptance criteria set by the developer (mean ± 2SD for both slope and R^2^), demonstrating robust inter-operator variability.

The LOD for this assay was thus determined to be 10 copies, with a 2.08% false negative rate for an individual replicate. No false negatives were observed for assays run in triplicate, with positive detection defined as a minimum of 2 of the 3 replicates in a single assay showing the presence of *VSV-G*. A single false positive in a negative control sample (NTC) was also observed in this dataset (Figure 4), putting the false positive rate for this dataset at 2.08% (Table S2). This results in a total false positive rate of 1.28% in human DNA, with or without DMSO (26 assays, 3 replicates/assay; full data not shown). The false positive rate in nuclease-free water is 1.67% (1 false positive in 20 assays, 3 replicates/assay; full data not shown). Our total false positive rate for all negative control samples, regardless of diluent, is currently 1.45%. No assays showed more than 1 of 3 replicates, with a C_q_ value in a negative control.

**Figure 5:**
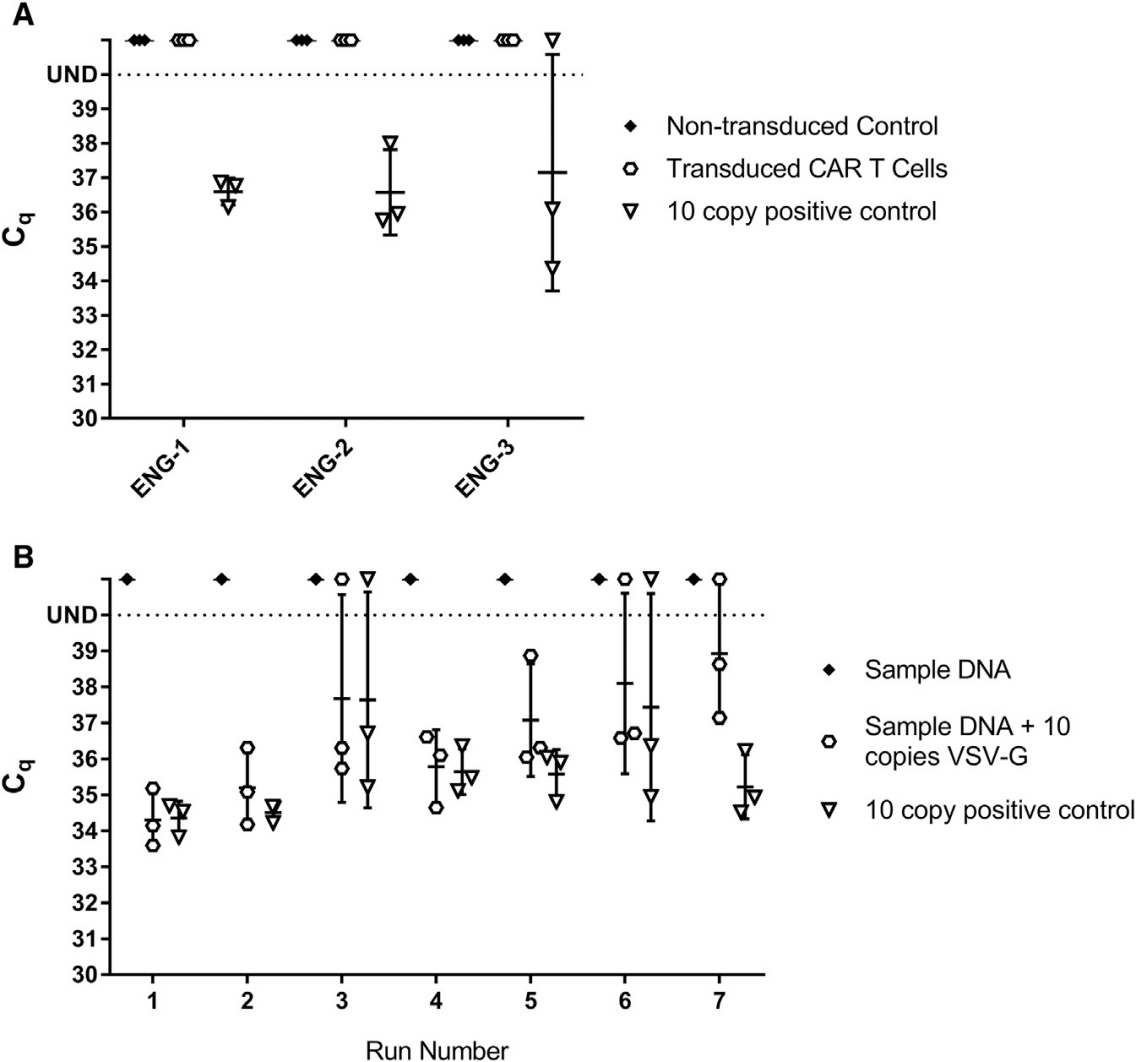
Detection of *VSV-G* DNA in Clinical Product Test Samples (A) C_q_ values for non-transduced control samples and transduced CAR T cell samples compared with the C_q_ values of the 10 copy positive control in the standard curve for each respective assay. (B) C_q_ values for sample DNA alone, sample DNA with 10 copies of *VSV-G* plasmid DNA spiked in, and the 10 copy positive control on the same assay (n = 7). Error bars represent SD.

False positive rates are typically higher in samples from cells that have been transduced with lentivirus due to residual presence of residual *VSV-G* plasmid DNA from the viral vector production. We thus tested transduced T cell samples from 3 separate engineering runs of a CAR T production process intended for use in manufacturing phase I clinical materials in our facility to determine the false positive rate from our sample preparation. The first 2 engineering runs utilized a non-GMP viral vector batch that lacked full purification and testing for residual DNA. The 3^rd^ engineering run utilized the fully GMP-compliant vector intended for patient use. The Certificate of Analysis from that vector production stated there was no evidence of RCL, but there was a residual *VSV-G* plasmid DNA load of 6.7 × 10^4^ copies/μL, despite treatment with benzonase during vector purification. For each engineering run, the cells were cultured for 9 days. Transduction with the viral vector occurred 1 day after the start of culture and T cell activation. Full media exchanges were performed on day 5, media feed on day 6, and media demi-depletion on day 7. Samples of 1 × 10^6^ cells were collected on day 9 and washed 3 times with 1 mL of Dulbecco’s phosphate buffered saline (DPBS) prior to DNA isolation for RCL testing. A non-transduced control was also cultured from the same donor cells side by side for comparison. For each of these samples, *VSV-G* DNA was undetectable after 40 cycles of qPCR in both the sample and untransduced control (Figure 5A). In contrast, *VSV-G* was detectable in samples that underwent an identical DNA purification process when 10 copies of VSV-G plasmid DNA were spiked in Figure 5B. One of the positive spike-in control replicates in ENG-3 was undetectable; however, the sample would have been considered positive for *VSV-G*, with 2 of the 3 replicates being detectable. Therefore, our method of DNA preparation does not inhibit *VSV-G* DNA detection, but does reduce residual *VSV-G* plasmid DNA used in vector manufacturing to a degree that it is undetectable in a sample.

## Discussion

The current standard for RCL testing is the use of a cell-based assay using a permissive cell line to allow for expansion of low-level virus followed by detection of viral proteins. These assays typically require 6 weeks or more before results are available. The FDA currently allows the use of qPCR-based assays for quick determination of RCL presence in order to have fresh infusion of the cell product. With the increasing reliance on rapid (PCR) testing for RCL, it is important to develop assays that are reproducible, accurate, and have robust sensitivity while maintaining a low rate of false positives in order to ensure adherence to patient safety standards.

We demonstrate here that we have created a qPCR assay that is linear over several decades of target sequence concentration, specific for the target sequences (low false positive rate of 1.45% in the absence of target sequence), reproducible within and between operators and sensitive enough to reliably detect 10 copies of *VSV-G* target sequence per reaction. We have also demonstrated that the presence of genomic DNA reduces the sensitivity of the assay (compared to assays run in nuclease-free water with plasmid only) but that the sensitivity of the assay can be recovered by including 3% DMSO in the PCR reaction. The assay thus meets the primary requirements for an analytical test, as outlined in FDA guidance documents.^17^, ^18^

Assays using this procedure will be considered valid if at least 2 of the 3 replicates for all negative control samples (NTC and untransduced control for the cell product) have undetectable *VSV-G*. If one of the replicates has detectable *VSV-G*, the C_q_ value must be greater than the mean C_q_ of the 10 copies/reaction positive control. The standard curve must have a slope of −3.3422 ± 0.2354 and R^2^ ≥ 0.99. The limit of detection for this assay is 10 copies, with a 97.92% detection rate. Due to the presence of a 1.45% false positive rate, cell product samples testing for RCL must have at least 2 of the 3 replicates as undetectable, and the third replicate, if detectable, must be greater than the mean C_q_ of the 10 copies/reaction positive control to be considered negative for RCL. A product with 1 of the 3 replicates being undetectable and the other 2 replicates with C_q_ values less than or equal to the mean C_q_ of the 10 copies/reaction positive control must be considered a preliminary positive for RCL and sent to a third party for a cell-based RCL test. Our data from transduced CAR T samples show that our culturing of the cells and triplicate washing of the samples are sufficient to reduce residual *VSV-G* DNA from the vector below the levels that are detectable by this assay.

## Materials and Methods

### Primer and Hydrolysis Probe Design

Primer/probe sets were designed using the Primer3 online software from the Whitehead Institute for Biomedical Research. Three potential primer/probe sets were further analyzed using the online Primer Stat Sequence Manipulation Suite and blasted against the human genome on the NCBI nblast online tool. None of the primer/probe sets showed homology to any part of the published human genome, and 2 of the 3 sets showed no potential self-annealing through Primer Stat. Those 2 sets were ordered (primers from IDT; probe from Applied Biosystems) and assayed for amplification of a *VSV-G*-containing plasmid. Only 1 of the 2 sets showed amplification of a single band at 59°C and 60°C using TaqPath qPCR Master Mix, CG (Applied Biosystems). The other set showed no amplification at any temperature assayed. The primer/probe set chosen for further assay development is as follows.

Forward primer: 5′-AGGGAACTGTGGGATGACTG-3′
Reverse primer: 5′-GAACACCTGAGCCTTTGAGC-3′
Hydrolysis probe: 5′-6FAM-GAACACCTGAGCCTTTGAGC-MGBNFQ-3′

### qPCR Protocol

pCMV-G was a gift from City of Hope. The plasmid was expanded using transformation of *E. coli* and purification of plasmid using a Machery-Nagel Low-Endotoxin Maxi Prep kit. The resulting plasmid was quantified using a nanophotometer (Implen) and diluted to 1 × 10^8^ copies/μL in low EDTA Tris-EDTA (TE) buffer and aliquoted and stored at −20°C for use as the *VSV-G* standard in the qPCR standard curves. All standard curves were based on a 10-fold dilution series. When preparing the standard curve samples, the first dilution from the stock 1 × 10^8^ copies/μL solution was 1:12.5 to produce a 1 × 10^8^ copies/reaction solution that accounts for the 12.5 μL of standard solution to be added for each reaction replicate. A 10-fold dilution series was then performed down to a solution of 1 × 10^1^ (10) copies/reaction. The diluent for the standard curves was either water, 8 ng/μL control human DNA, or 8 ng/μL control human DNA, with DMSO added to the reaction master mix for a final concentration of 3% (v/v). The control human DNA for the standard curve dilution background was purchased from Genscript and diluted to 8 ng/μL with UltraPure DNase/RNase-Free Distilled Water (Invitrogen). The NTC for each standard curve was the diluent for the standard curve in the absence of plasmid. QC samples for positive controls were generated by performing a second 10-fold dilution series, starting with a 500,000 copies/reaction sample. The 50 copies/reaction sample was then diluted into the individual QC limit of detection controls. Diluents for the generation of the QC samples were paired with the diluents for the standard curves.

Each of the NTC, standard curve solutions, and QC controls were analyzed on a single plate in triplicate using a standard curve TaqMan assay standard protocol on the QuantStudio 7Flex, with a reaction volume of 25 μL, annealing temperature of 60°C, and 40 cycles using TaqPath qPCR Master Mix, CG (Applied Biosystems).

### CAR T Transduction and Culture

A leukapheresis product (STEMCELL Technologies) from a healthy donor was positively selected for CD4^+^ and CD8^+^ cells on the CliniMACS Prodigy (Miltenyi). The selected cells were cultured on the Prodigy in TexMACS GMP Medium (Miltenyi) with 3% (v/v) human AB serum (Valley Biomedical) and 200 IU/mL rhIL-2 (Prometheus Therapeutics and Diagnostics) using the Miltenyi T Cell Transduction Process for the CliniMACS Prodigy. Cells were activated on day 0 using GMP T cell TransAct (Miltenyi). Cells were transduced on day 1 with the MSCV-CAR1922-WPRE lentivirus (Lentigen) at an MOI of 40. Cells received a full culture wash of fresh medium on day 5, culture feed by medium addition on day 6, and culture feed by medium demi-depletion on day 7. Samples were removed from the culture on day 9 for RCL analysis prior to harvest on the same day.

### Sample Preparation

DNA was extracted from CAR T cells and their untransduced controls using the PureLink Genomic DNA Mini Kit (Invitrogen). For each sample preparation, 1 × 10^6^ cells were washed 3 times with 1 mL of 1X DPBS. The cell pellet was resuspended in 200 μL of 1X DPBS. 20 μL of proteinase K and 20 μL of RNase A were added to the cell suspension, and the samples were incubated for 2 min at room temperature. 200 μL of PureLink Genomic DNA Lysis/Binding Buffer was added to each cell suspension, and cells were incubated for 10 min at 56°C. 200 μL of 200-proof ethanol was added, and the lysate was loaded onto PureLink Spin Columns. The column was then washed once with 500 μL of Genomic Wash Buffer 1, followed by a wash with 500 μL of Genomic Wash Buffer 2. The column was centrifuged for 5 min at 10,000 × *g* after the addition of Genomic Wash Buffer 2 in order to more thoroughly remove all ethanol contaminants from the column. The DNA was eluted from the column in a duplicated elution step with 25 μL, providing a final sample volume of 50 μL. DNA was quantified on an Implen Nanophotometer, and A260/A280 and A260/A230 values were recorded as a representation of sample purity. The DNA was diluted to 8 ng/μL in UltraPure DNase/RNase-Free Distilled Water (Invitrogen). Samples were stored at −20°C for 1 week to 3 months prior to testing with the qPCR protocol.

### Data Analysis

Quantification cycle (C_q_) values were determined automatically by the QuantStudio software. Samples that lacked expression above the quantification threshold set by the QuantStudio were considered to have no detection (UND = undetectable) of *VSV-G* DNA. Standard curves were generated using Microsoft Excel. Copy number quantities for the standard curve samples were converted to Log10 of the copies present in the reaction and plotted on the x axis. The y axis values were plotted as the C_q_ for each replicate. Linear trend lines were then added to determine slope and R^2^ of each standard curve. All graphs are scatterplots showing individual data points, with the error bars showing the mean and SD. Statistics for graphs comparing 2 groups (Figures 1C and 1D) were done using the Mann-Whitney t test. Statistics for graphs comparing 3 groups (Figures 3A and 3B) were done using the Kruskall-Wallis one-way ANOVA test.

## Author Contributions

Conceptualization, L.M.S., D.L.D.; Methodology, L.M.S.; Investigation, L.M.S.; Formal Analysis, L.M.S.; Validation, B.M.K., R.J.A., M.F.L.; Visualization, L.M.S.; Writing – Original Draft, L.M.S.; Writing – Review and Editing, D.L.D.; Supervision, D.L.D.

## Supplemental Information

Document S1. Tables S1 and S2

Document S2. Article plus Supplemental Information
