# Detection of Replication Competent Lentivirus Using a qPCR Assay for VSV-G

**DOI:** 10.1016/j.omtm.2018.02.007

**Published:** 2018-02-22

**Authors:** Lindsey M. Skrdlant, Randall J. Armstrong, Brett M. Keidaisch, Mario F. Lorente, David L. DiGiusto

(Molecular Therapy: Methods & Clinical Development *8*, 1–7; March 16, 2018)

## Main Text

Following the publication of this article, the authors noted that the hydrolysis probe sequence was written incorrectly. The correct hydrolysis probe sequence is 5’-6FAM-ATTGGACATGGTATGTTGGACTCCG-MGBNFQ-3’.

