# Supplementary material for: Detection of Replication Competent Lentivirus Using a qPCR Assay for VSV-G
Source: Mol Ther Methods Clin Dev. 2017 Sep 21;8:1–7. doi: 10.1016/j.omtm.2017.09.001 (PMC5633339; doi:10.1016/j.omtm.2017.09.001)
Supplement: Document S1. Tables S1 and S2 [file mmc1.pdf]

OMTM, Volume *8*

Supplemental Information

Detection of Replication Competent Lentivirus Using a qPCR Assay for VSV-G

Lindsey M. Skrdlant, Randall J. Armstrong, Brett M. Keidaisch, Mario F. Lorente, and David L. DiGiusto

Supplemental Table 1. Limit of Detection in Water vs. DNA

	Water Diluent	DNA diluent
10 copies	100.00%	83.33%
8 copies	83.33%	75.00%
6 copies	83.33%	66.67%
5 copies	76.19%	61.90%
4 copies	66.67%	58.33%
3 copies	66.67%	33.33%
NTC	0.00%	0.00%

Supplemental Table 1. Limit of Detection for VSV-G in nuclease-free water and control human DNA. Percentage of wells that had detectable VSV-G within 40 cycles for 10, 8, 6, 5, 4, and 3 copies/reaction and no template control (NTC).

Supplemental Table 2. LOD in DNA + 3% DMSO

	Water Diluent	DNA diluent	DNA diluent + 3% DMSO
10 copies	100.00%	83.33%	97.92%
8 copies	83.33%	75.00%	83.33%
6 copies	83.33%	66.67%	79.17%
5 copies	76.19%	61.90%	68.75%
4 copies	66.67%	58.33%	68.75%
3 copies	66.67%	33.33%	52.08%
NTC	0.00%	0.00%	2.08%

Supplemental Table 2. Limit of Detection for VSV-G in nuclease-free water, control human DNA, and control human DNA with a final 3% (v/v) DMSO reaction concentration. Percentage of wells that had detectable VSV-G in a DNA diluent with 3% DMSO within 40 cycles for 10, 8, 6, 5, 4, and 3 copies/reaction and no template control (NTC). Data for nuclease-free water and control human DNA was repeated from Supplemental Table 1 for comparison.
